# Dermatological Manifestations in Patients With SARS-CoV-2: A Systematic Review

**DOI:** 10.7759/cureus.9446

**Published:** 2020-07-28

**Authors:** Abdulelah Almutairi, Mohammed Alfaleh, Muath Alasheikh

**Affiliations:** 1 College of Medicine, King Saud University, Riyadh, SAU

**Keywords:** dermatological, manifestations, symptoms, sars-cov-2, covid 19

## Abstract

Severe acute respiratory syndrome coronavirus 2 (SARS-CoV-2) has been initially defined as a disease of the respiratory tract; however, with the increasing number of patients and announcing that the virus became a pandemic, new systemic clinical manifestations are observed, including dermatological manifestations. However, the identification and characteristics of these manifestations are still controversial. This review article aims to evaluate the medical literature and explore the dermatological clinical manifestations in patients with SARS-CoV-2. The literature was reviewed through MEDLINE®, Ovid, PubMed®, and Embase®. Searching terms included were a combination of "dermatological" OR "skin" AND "symptoms" OR "manifestations" AND "SARS-CoV-2". The following step was filtering the results to include only original research studies investigating the different types of skin and dermatological clinical manifestations in patients with SARS-CoV-2. A total of 879 studies were retrieved. Following the exclusion of studies on animals and including only studies on humans, 32 studies emerged. Altogether, seven studies were identified as eligible, covering 555 patients with SARS-CoV-2 who had dermatological symptoms. Three studies were retrospective, two studies were prospective, and two studies were case series. Different types of dermatological lesions can occur in patients with SARS-CoV-2, most commonly erythema, urticaria, and varicella-like rash. Dermatological manifestations with SARS-CoV-2 can be misdiagnosed with other conditions. Further studies with robust design are needed.

## Introduction and background

Coronaviruses are defined as a class of viruses that commonly lead to mild to moderate respiratory tract infections [1]. Moreover, in the last few years, there were some mutations that occurred in coronaviruses leading to transmission from animals to humans [2]. Furthermore, the virulence of the virus has increased, leading to increased mortality. Examples of these viruses are the Middle East respiratory syndrome-related coronavirus (MERS-CoV), severe acute respiratory syndrome coronavirus (SARS-CoV), and the recently explored severe acute respiratory syndrome coronavirus 2 (SARS-CoV-2) [3].

SARS-CoV-2 virus has been primarily identified in Wuhan city in China, in November 2019 [4]. The transmission rate of the virus started to increase rapidly and progressively till being announced as a pandemic by the WHO in February 2020 [5].

Signs and symptoms of the new viral infection might range from an absence of symptoms to severe and sometimes, life-threatening condition [6]. At the beginning of this wave of infection, it was thought that SARS-CoV-2 affects only the respiratory tract; however, with the increasing number of new cases globally, other systemic symptoms have been reported, which varied in severity [7]. 

One of these systemic symptoms is dermatological manifestations [8]. Some patients with SARS-CoV-2 were observed to have some cutaneous symptoms such as urticaria spreading over the body, erythematous rash, skin vesicles, similar to chickenpox infection [9]. These dermatological symptoms were commonly reported all over the body, particularly over the trunk [10]. Also, patients with SARS-CoV-2 complained of itching of varying severity. However, there is still a debate on these symptoms and whether there are other symptoms identified in patients with SARS-CoV-2 [11].

Our review aims to examine the current medical literature to explore the different types of dermatological clinical manifestations in patients with SARS-CoV-2. 

## Review

Methodology and search strategy

This systematic review of the literature was performed in compliance with the Preferred Reporting Items for Systematic Reviews and Meta-Analyses (PRISMA) checklist recommendations for systematic review and meta-analysis [12]. This systematic review was carried out through searching electronic databases to include eligible studies in four databases, including MEDLINE®, PubMed®, Ovid, and Embase®. 

Searching terms included "dermatological" OR "skin" AND "symptoms" OR "manifestations" AND "SARS-CoV-2". All the titles, as well as abstracts that emerged from this exploration, were reviewed completely to prevent missing any eligible studies. The results were then filtered to include only original research studies examining the different types of skin and dermatological clinical manifestations in patients with SARS-CoV-2. Additionally, all study designs from different countries were included. Only studies written in the English language were listed as related studies, which can be further assessed in the second step. 

Eligibility criteria

**Figure 1:**
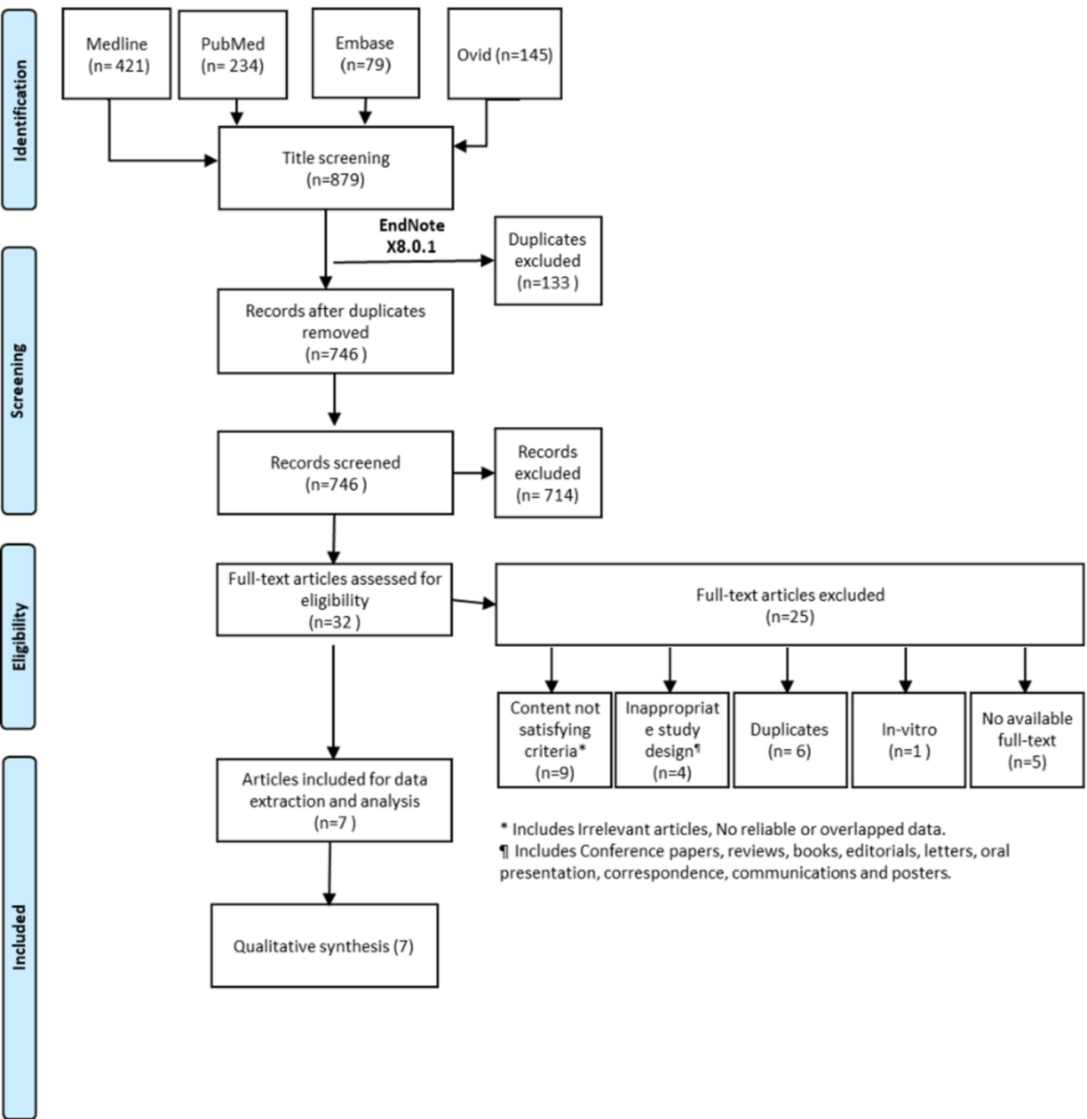
Search strategy through four databases to select eligible studies

Following this stage, the inclusion criteria to choose the studies that will be recognized in the systematic review were determined. Abstracts were reviewed manually to determine the appropriate abstracts to be considered. The inclusion criteria included discussing enough data on the dermatological symptoms with SARS-CoV-2. Moreover, only studies done among adult patients were included. Furthermore, references of the chosen studies were assessed to distinguish any related studies. Lastly, the required data sets were collected from the final record of eligible studies and summarized. Studies were eliminated in case of in vitro or animal involvement, overlapped or incomplete data, and unavailability of full-text studies or inappropriate study design. Entire details on the search strategy are shown in Figure 1.

Data review and analysis

The first step included a preparatory review, a specially designed excel sheet was used for data extraction. Chosen data from eligible studies were then reviewed through the excel sheet. Any studies published by one research group examining alike variables were evaluated for any potential duplication. Cochrane, a quality assessment tool, was used to evaluate the quality of the included clinical studies [13]. Data were then statistically expressed in terms of frequencies (number of cases) and percentages for categorical variables. Mean, standard deviations, medians, and interquartile ratios were used to represent the numerical variables. All statistical calculations were performed by IBM SPSS version 26 (Statistical Package for the Social Science; IBM Corp, Armonk, USA).

Results

After searching the abstracts and reviewing the eligibility criteria in identified potential abstracts, a total of seven studies [14-20] were considered as eligible to be included in the present systematic review, covering a total of 555 patients with SARS-CoV-2 who had dermatological symptoms in the form of skin lesions. Out of the seven studies, two studies were prospective [14, 16], three studies were retrospective [17, 19, 20], and two studies were case series [15, 18]. 

Additionally, All the included studies considered the objective of describing the dermatological manifestations of patients with SARS-CoV-2 with varying severity levels in different countries. Due to the scarcity of data and lack of experience with the newly identified virus, all study designs were included [14-20].

Furthermore, all the studies identified the types of dermatological manifestations in patients. Erythema and urticaria were common in four studies [14, 17- 19], varicella-like lesions were defined in three studies [15, 17, 19], while other types of rash were identified in four studies [16-18, 20]. The included studies are discussed in detail in Table 1.

**Table 1:** Data extracted from eligible studies including dermatological symptoms

Authors	Year	Study design	Sample size	Type of dermatological symptom	Objective	Result
Galván Casas et al. [14]	2020	prospective	375	Erythema, urticaria, vesicular eruptions	To describe the dermatological manifestations of SARS-CoV-2 disease and to correlate them to other clinical findings.	Lesions may be assigned as acral areas of erythema with vesicles or pustules (pseudo-chilblain) (19%), urticarial lesions (19%), other vesicular eruptions (9%), maculopapular eruptions (47%) and livedo or necrosis (6%). Vesicular eruptions develop early in the progression of the disease (15% before other symptoms). The pseudo-chilblain pattern developed late in the progression of the SARS-CoV-2 disease (59% following other signs), while the remaining symptoms appear with other manifestations of SARS-CoV-2. The severity of SARS-CoV-2 shows a gradient from the less severe disease in acral lesions to most severe in the latter groups.
Marzano et al. [15]	2020	case series	22	Varicella-like exanthem	To describe dermatological lesions in the Italian population.	This was varicella-like exanthem as a specific SARS-CoV-2 associated; its typical features are constant trunk involvement, usually scattered distribution, and mild/absent pruritus, the latter being in line with most viral exanthems but unlike true varicella. Lesions generally appear three days after systemic symptoms and disappear upon eight days, without leaving scarring.
Landa et al. [16]	2020	prospective	6	Multiple lesions similar to chilblains	To describe skin lesions in six patients presenting with SARS-CoV-2 mild symptoms	The six patients had multiple skin lesions, especially on the toes, soles, fingers, extremities, and heel, similar to chilblains. The patients were with mild coronavirus symptoms. Very few refer to fever, cough, or congestion three to four weeks earlier, and some patients had high-risk contacts. Two of the six patients had a positive test weeks earlier. The lack of confirmatory testing did not allow an association of these types of lesions with SARS-CoV-2. These lesions could be a delayed manifestation of SARS-CoV-2. The lesions developed weeks after reaching the peak of infections. This is supported by some of the patients who reported similar manifestations or higher risk individuals (sick persons or health workers) weeks before the occurrence of skin lesions. These lesions can be an antigen-antibody immunological process when the viral load and infectivity are low.
Bouaziz et al. [17]	2020	retrospective	14	Exanthema, urticaria, varicella-like rash, livedo, necrosis	To describe the characteristics of skin lesions in patients with SARS-CoV-2 infection.	Skin symptoms started a few days after the first SARS-CoV-2 general symptoms. Inflammatory lesions were reported in seven patients: exanthema (four patients), chickenpox like vesicles (two patients), cold urticaria (one patient). Vascular lesions were reported in seven patients: violaceous macules with "porcelain-like" appearance (one patient), livedo (one patient), nonnecrotic purpura (one patient), necrotic purpura (one patient), chilblain appearance with Raynaud's phenomenon (one patient), chilblain (one patient), eruptive cherry angioma (one patient).
Alramthan et al. [18]	2020	case report	2	Red-purple papules, erythema	To describe cutaneous lesions in two patients with SARS-CoV-2 in Qatar.	Two cases of SARS-CoV-2 presenting with cutaneous lesions. A 27-year-old and 35-year-old previously healthy females with a chief complaint of a skin rash. Physical examination revealed red-purple papules on the dorsal aspect of fingers bilaterally. The 35-year-old female additionally had diffused erythema in the subungual area of the right thumb.
Recalcati et al. [19]	2020	retrospective	88	Erythematous rash, urticaria, varicella-like a rash	To investigate the type of cutaneous lesions in patients with SARS-CoV-2 in Italy.	Eighteen patients (20.4%) developed cutaneous manifestations. Eight patients developed cutaneous involvement at the onset, ten patients after the hospitalization. Cutaneous manifestations were erythematous rash (14 patients), widespread urticaria (three patients) and chickenpox-like vesicles (one patient). The trunk was the main involved region. The itching was low or absent, and usually, lesions healed in a few days. There was not any correlation with the disease’s severity. Skin manifestations are similar to cutaneous involvement occurring during common viral infections.
Joob et al. [20]	2020	retrospective	48	A skin rash with petechiae confused with dengue	To describe skin lesions in SARS-CoV-2 patients in a hospital in Thailand.	One patient out of the 48 patients had a skin rash with petechiae. Because dengue is very common in Thailand, petechiae rash is a frequent clinical manifestation in dengue. The patient had thrombocytopenia. Dengue was misdiagnosed in the beginning. There was no image, and a biopsy is not routinely done for dengue diagnosis in Thailand. The patient was initially misdiagnosed as dengue, which has led to a delayed diagnosis There is a chance that a patient with SARS-CoV-2 can primarily have a skin rash that can be misdiagnosed as another common disease.

Discussion

The novel SARS-CoV-2 virus was identified in late 2019, with minimal knowledge and experience regarding its diagnosis and treatment until present [4]. Although the symptoms of SARS-CoV-2 are mainly affecting the respiratory tract, other systemic features have been identified [13]. Dermatological manifestations, such as urticaria, erythema and rash, were also identified in some patients with SARS-CoV-2 in different countries. However, the description of these manifestations and their correlation to SARS-CoV-2 is still controversial [18].

The present review evaluated the description and characters of dermatological manifestations in patients with SARS-CoV-2. It has been shown that different types of cutaneous lesions occur in patients with SARS-CoV-2, even in patients with mild respiratory symptoms. Hence, the misdiagnosis of dermatological lesions in SARS-CoV-2 is common.

Furthermore, the most frequent types of lesions were varicella-like rash and rash resembling recent viral infection, in addition to urticaria and erythema. It is worth noting that these lesions might be itching or non-itching and are usually mildly severe in most patients, resolving at the end of the course of infection. Dermatological lesions can also develop at a later stage of the infection.

Erythema and urticaria in SARS-CoV-2 patients have been described in some studies. The most robust study design described dermatological manifestations in 375 Spanish SARS-CoV-2 patients. The study described the lesions as commonly occurring in the acral areas, with 19% having erythema in the form of pseudo-chilblain, which developed in later stages in the infection, and another 19% having urticaria lesions. Also, the dermatological lesions related to SARS-CoV-2 were severe in the acral area, with less severe lesions in other parts. Additionally, the maculopapular rash was also characteristic in Spanish patients with a prevalence of 47% [14].

Another study that had a retrospective design reviewed 14 patients and showed that dermatological lesions appeared at a later stage of the infection, with inflammatory lesions occurring in 50% of patients in the form of exanthema, chickenpox-like vesicles. Additionally, the study identified that the remaining 50% of patients had vascular lesions in the form of porcelain-like macules, livedo, necrotic purpura, chilblain, and eruptive cherry angioma [17].

Another study from Italy included 88 patients in a retrospective design and demonstrated that 20.4% of patients had cutaneous manifestations, with eight patients developed the dermatological symptoms at an earlier stage of the onset of the SARS-CoV-2 infection, while ten patients developed the lesions after hospitalization due to SARS-CoV-2 due to severe infection. Importantly, there was no correlation between the occurrence of dermatological lesions and the severity of SARS-CoV-2 infection, where some patients had dermatological lesions without developing respiratory symptoms for SARS-CoV-2. The study also highlighted that the lesions are resembling those occurring in common viral infections [19]. This finding was also supported by a varicella-like rash observed by two studies [15, 17].

It is worth to mention that other frequent conditions can misdiagnose dermatological lesions occurring in patients with SARS-CoV-2. In Thailand, one study described one out of 48 SARS-CoV-2 patients who had a rash with petechiae that was misdiagnosed with dengue disease, especially with the absence of routine biopsy in this setting [20].

The included studies also had some limitations; most of the included studies were performed in one center, which may affect the external validity of outcomes. Also, the sample size in each study was relatively small, which could reduce the reliability of their outcomes. These limitations should be considered in future studies.

Finally, this is considered the first systematic review to identify the dermatological manifestations occurring in patients with the novel SARS-CoV-2 infection. This should be a guide for clinicians to prevent the misdiagnosis of these manifestations with other dermatological conditions.

## Conclusions

Dermatological lesions are frequent in patients with SARS-CoV-2, especially erythema, urticaria, and varicella-like rash. Differential diagnosis should be thoroughly considered before deciding that the present rash is related to SARS-CoV-2 infections. Till present, the rash is not correlated to the severity of SARS-CoV-2 infection, which needs to be confirmed with studies with more robust design and larger sample sizes. These findings should be considered by clinicians working with patients with SARS-CoV-2 in order not to misdiagnose the occurrence of dermatological lesions, which may delay therapy or increase the risk of complications.
